# Novel proton conducting core–shell PAMPS-PVBS@Fe_2_TiO_5_ nanoparticles as a reinforcement for SPEEK based membranes

**DOI:** 10.1038/s41598-021-84321-7

**Published:** 2021-03-01

**Authors:** Parisa Salarizadeh, Mehran Javanbakht, Mohammad Bagher Askari, Khadijeh Hooshyari, Morteza Moradi, Hossein Beydaghi, Mohadese Rastgoo-Deylami, Morteza Enhessari

**Affiliations:** 1 High-Temperature Fuel Cell Research Department, Vali-e-Asr University of Rafsanjan, Rafsanjan, Iran; 2 Department of Chemistry, Amirkabir University of Technology, 1599637111 Tehran, Iran; 3 Department of Physics, Faculty of Science, University of Guilan, P.O. Box, 41335-1914 Rasht, Iran; 4 Department of Applied Chemistry, Faculty of Chemistry, Urmia University, Urmia, Iran; 5 Department of Chemical Engineering, Faculty of Engineering, Shahid Chamran University of Ahvaz, Ahvaz, Iran; 6 Graphene Labs, Istituto Italiano di Tecnologia, via Morego 30, 16163 Genoa, Italy; 7 Department of Physics, Sharif University of Technology, 11155-9161 Tehran, Iran; 8 Department of Chemistry, Naragh Branch, Islamic Azad University, Naragh, Iran

**Keywords:** Electrochemistry, Fuel cells, Nanoparticles

## Abstract

In this study, new nanocomposite membranes from sulfonated poly (ether ether ketone) (SPEEK) and proton-conducting Fe_2_TiO_5_ nanoparticles are prepared by the solution casting method. Sulfonated core–shell Fe_2_TiO_5_ nanoparticles are synthesized by redox polymerization. Therefore, 4-Vinyl benzene sulfonate (VBS) and 2-acrylamide-2-methyl-1-propane sulfonic acid (AMPS) are grafted on the surface of nanoparticles through radical polymerization. The different amounts of hybrid nanoparticles (PAMPS@Fe_2_TiO_5_ and PVBS@Fe_2_TiO_5_) are incorporated into the SPEEK matrix. The results show higher proton conductivity for all prepared nanocomposites than that of the SPEEK membrane. Embedding the sulfonated Fe_2_TiO_5_ nanoparticles into the SPEEK membrane improves proton conductivity by creating the new proton conducting sites. Besides, the nanocomposite membranes showed improved mechanical and dimensional stability in comparison with that of the SPEEK membrane. Also, the membranes including 2 wt% of PAMPS@Fe_2_TiO_5_ and PVBS@Fe_2_TiO_5_ nanoparticles indicate the maximum power density of 247 mW cm^−2^ and 226 mW cm^−2^ at 80 °C, respectively, which is higher than that of for the pristine membrane. Our prepared membranes have the potential for application in polymer electrolyte fuel cells.

## Introduction

The proton exchange membrane (PEM) plays an important role in proton transport from the anode to the cathode side of the polymer electrolyte membrane fuel cells (PEMFCs). By considering this fact, this part of the fuel cell has always attracted the researchers’ attention^1,2^. As an essential component of PEMFCs, PEMs must have outstanding characteristics such as high proton conductivity, desirable physicochemical and thermal properties, dimensional stability, and low cost^3^. Up to now, perfluorosulfonic acid polymers such as the Nafion membrane have been widely utilized as PEM due to their outstanding properties including high proton conductivity under humid conditions and long-term stability^4^. However, some drawbacks such as low proton conductivity at operating temperatures above 80 °C because of water evaporation, high fuel permeability, and high-cost limited their applications^5,6^. Accordingly, the development of low-cost alternative materials with superior properties remains an attractive and promising demand. To solve this concern, researchers have focused on new methods such as blending, cross-linking, and nanocomposites based on polymers such as sulfonated poly(ether sulfone) (SPES)^7^, sulfonated poly (phthalazinone ether ketone) (SPPEK)^8^, sulfonated poly(ether ether ketone) (SPEEK)^9^, poly(vinylidene fluoride-co-hexafluoropropylene) (PVdF-HFP)^10^, and poly polybenzimidazole (PBI)^11^.

Among these alternative polymers, one of the promising candidates is SPEEK, which has desirable and controllable properties to use as the electrolyte of PEMFCs^12,13^. A SPEEK-based membrane has been reported to possess high proton conductivity, good thermal and mechanical stabilities, low cost, and low fuel crossover, which is due to their narrow and less connected hydrophilic channels^14^. The SPEEK-based membrane has a phase-separated morphology at a hydrated state, which gives rise to a connected network of transporting channels for proton transfer^15,16^. It was found that the degree of sulfonation (DS) has the most important effect on the proton conductivity of SPEEK. At a low DS, the hydrophilicity of the SPEEK is too low for acceptable proton transfer by water molecules, whereas a prepared membrane with a too high DS presents an excessive swelling with unacceptable mechanical stability^17^. Besides, the new approach among all the strategies to enhancing membrane performance at operating temperature is to incorporate nano-sized inorganic fillers into the polymeric backbone to fabricate nanocomposite membranes^18^. As mentioned, many researchers have been interested in inorganic–organic composite systems due to the probability of achieving demanded characteristics that do not exist in pristine membranes. During the past years, some inorganic additives, such as sulfonated graphene oxide^19^, sulfonated tungsten trioxide^10^, sulfonated silica^20^, sulfonated TiO_2_^21^, and perovskite-structure nanoparticles have been successfully introduced into the polymeric backbone by our group and exhibited their considerable potential for use in a PEM. The ytterbium/yttrium^22^, lanthanum cerium oxide^23^, and iron titanate (Fe_2_TiO_5_)^24,25^ are some of the perovskite-structure nanoparticles that have been investigated by our group.

In recent years, perovskite nanoparticles have been received great attention for use in PEMFCs because of their improved chemical, mechanical, and thermal stability^26^. The proton transfer in perovskite nanoparticles is due to hydrogen bonds created between their neighboring lattice oxygen which this bonding facilitates the proton conductivity of the prepared PEM. Furthermore, various hydrogen bonding sites can be accessible due to the inter- or intra-molecular interactions formed between perovskite nanoparticles and organic polymer matrixes which cause to achieve a desirable water uptake and contribute to enhancing proton conductivity. Apart from the benefits mentioned for the inorganic additives, the specific interactions between the inorganic and organic groups also resulted in superior chemical stability^27^. Totally, despite the considerable development in the efficiency of PEMs membranes some technical concerns still require solutions. In this regard, the poor interfacial interaction between inorganic additives and an organic polymer remains a critical problem showing a noticeable impact on the proton conductivity and hence the total efficiency of the membrane.

For solving this problem, surface modification of nanoparticles has attracted considerable attention as an efficient solution to enhance nanoparticle performance for various applications such as PEMFCs^28–30^. Following surface modification of nanoparticles, excess functional groups are also capable of binding with different chemical species. Zhang et al.^31^ prepared nanocomposite membranes by incorporating synthesized PWA (phosphotungstic acid) –NH_2_-HMS (hollow mesoporous silica) nanoparticles in polyether sulfone-polyvinylpyrrolidone (PES-PVP). In this study, the optimum sample with 10 wt% PWA-NH_2_-HMS showed a proton conductivity and power density of 175 mS cm^−1^ and 420 mW cm^−2^, respectively. Qiu et al.^32^ introduced poly(2,5-benzimidazole)-grafted graphene oxide into SPEEK polymeric backbone and observed that membranes showed improved water uptake, swelling ratio, and proton conductivity. The prepared membrane exhibited the proton conductivity of 7.5 mS cm^−1^, which was four times higher than that of SPEEK. Also, the optimum value of power density (831.06 mW cm^−2^) was achieved at 80 °C and 90% relative humidity.

It’s well-known that by incorporating organic modifiers on the surface of nanoparticles the ability of dispersion of the nanoparticles has improved. The most likely explanation for this phenomenon is that by attaching organic modifiers such as aminopropyltrimethoxysilane (APTMS)^8^ and poly sulfonic acid^21,33^ on the surface of nanoparticles, the hydrophilicity of nanoparticles is restricted and the dispersion ability increased which lead to the more uniform and durable membrane. In this regard, surface modification of Fe_2_TiO_5_, which is a perovskite-type oxide nanoparticle, has found promising attraction in PEMFCs as a proton conductor nanoparticle. In our previous investigation, Fe_2_TiO_5_ nanoparticles modified by silane coupling agent was successfully synthesized and applied as PEMs^8^. Besides, 3-aminopropyltriethoxysilane (APTES) was applied as an organic modifier and modified nanoparticles incorporated in SPPEK. Results showed that the surface-modified nanoparticles had a significant effect on the physicochemical properties of the prepared membrane. The nanocomposite membrane containing 3 wt% APTES modified nanoparticles showed the proton conductivity of 24 mS cm^−1^ and reached 149 mW cm^−2^ as maximum power density.

In this study, novel sulfonated core–shell Fe_2_TiO_5_ nanoparticles were prepared and then suspended in the SPEEK matrix. The poly acrylamide-2-methyl-1-propane sulfonic acid (PAMPS) and polyvinyl benzene sulfonate (PVBS) were used as a modifying agent. The modification of nanoparticles is non-bridging with other chemical reactions, so it can reduce the aggregation of the modified nanoparticles in organic solvents. The sulfonated groups of PAMPS and PVBS can increase proton hopping sites for proton transfer in the structure of the membrane, following by improving proton conductivity and power density. Because sulfonated groups exist in the structure of modifying agents, with an increasing number of hydrogen bonding in the structure of the nanocomposite membrane, the thermal and mechanical stability of the prepared membranes improves. The effect of the different amounts of the modified nanoparticles (0.5, 1, 1.5, 2, and 2.5 wt%) on different properties of the nanocomposite membranes were evaluated. In addition, the effect of adding the sulfonated nanoparticles on proton conductivity, dimensional stability, oxidative, thermal, and mechanical stabilities, and PEMFC performance was discussed.

## Experimental

### Materials

(3-Aminopropyl) triethoxysilane (APTES), purchased from Merck, was used as the surface modifier and the coupling agent. Triethylamine (TEA), as a catalyst for surface modification reaction, and ceric ammonium nitrate, as an oxidant and the initiator of the polymerization reaction, were purchased from Sigma-Aldrich. 4-Vinyl benzene sulfonate (VBS), 2-acrylamide-2-methyl-1-propane sulfonic acid (AMPS), and PEEK were also provided from Sigma-Aldrich. Fe_2_TiO_5_ nanoparticles were synthesized as demonstrated in our previous repor^8^. In addition, other chemicals used in this study were bought from Merck.

### Synthesis of core–shell Fe_2_TiO_5_ nanoparticles

In the beginning, Fe_2_TiO_5_ nanoparticles were dispersed uniformly in dry toluene by ultrasound to prepare a 1 wt% suspension. Next, 4 mL of APTES and 0.3 mL of TEA were mixed with 100 mL of Fe_2_TiO_5_ suspension and it was refluxed at 80 °C for 6 h. Then, to purify the amine-functionalized Fe_2_TiO_5_ nanoparticles, the resultant nanoparticles were washed with toluene and ethanol three times in each solvent by centrifugation and decantation. Finally, the obtained amine-functionalized Fe_2_TiO_5_ nanoparticles were dried at 80 °C for 24 h and named AFFT^25^.

Then, 0.1 g of the obtained AFFT nanoparticles was dispersed in 75 mL of 0.14 M nitric acid and was kept in an ultrasonic device for 15 min. Furthermore, 1 g of the monomer (VBS or AMPS) and 0.44 g of SDS were dissolved separately in 0.14 M nitric acid and were mixed with the nanoparticle suspension, respectively. Then, they were placed under ultrasonication for 15 min. To remove the dissolved oxygen in the solution, the suspension was purged with nitrogen under stirring for 30 min. Besides, 0.07 g of ceric ammonium nitrate was mixed with 5 mL of 0.14 nitric acid and quickly added to the obtained homogenous suspension. The reaction continued for 12 h to complete the polymerization reaction. After that, the functionalized nanoparticles were washed with a water/acetone mixture to remove unreacted monomers, and then they were dried in a vacuum oven. The obtained nanoparticles were named PAMPS@FT and PVBS@FT.

### Preparation of SPEEK

Direct sulfonation of PEEK was used for the synthesis of SPEEK. So, 2 g PEEK was mixed with 20 mL concentrated sulfuric acid for 1 h at ambient temperature to dissolve the polymer. Then, its stirring process continued for 4 h at 60 ºC to perform the reaction. After completion of the sulfonation reaction, the obtained mixture was cooled and added gradually to the huge amount of cold water to form SPEEK sediment. The obtained sediment was washed with distilled water to reach the neutral pH and then it was dried in an oven at 70 ºC. The titration method was utilized for the determination of the degree of SPEEK sulfonation. For this purpose, 0.2 g of the sulfonated polymers were added to 20 mL of 1 M NaCl solution and the obtained mixture was stirred for 24 h. Then, titration of the final solution was carried out with 0.02 M NaOH solution. The following equation was used for the calculation of the degree of sulfonation (DS).

1 $$DS = \frac{{10^{ - 3} \times 288.31 \times C_\text{NaOH} \times V_\text{NaOH} }}{{W - 0.081C_\text{NaOH} V_\text{NaOH} }} \times 100$$

where, C_NaOH_, V_NaOH_, and W refer to the concentration of NaOH solution (M), volume of consumed NaOH solution (mL), and the sample mass, respectively. Also, 288.31 and 81 are the molecular weight of a repeated unit of PEEK and that of the –SO_3_H group, respectively. The DS value was found to be about 68%.

### Preparation of membrane

The solvent casting method was used for the preparation of the nanocomposite membranes. SPEEK/PAMPS@FT and SPEEK/PVBS@FT nanocomposite membranes (named S/PA@FTx and S/PV@FTx, respectively) were synthesized with different weights of the nanoparticles, where x denoted the weight percentages of nanoparticles. So, 1 g SPEEK was added to 2.5 mL DMAC, and the obtained mixture was stirred for 1 h at 80 °C to solve completely the polymer. Then, different amounts of the nanoparticles (0.5, 1, 2, and 3 wt%) were mixed separately with 2.5 mL DMAC via ultrasound for 30 min and the obtained suspensions were mixed with the SPEEK solution. Then, the final mixture was stirred at ambient temperature for 30 min, and a film of the mixture was obtained by pouring it on glass via an automatic film applicator. The film was dried at ambient temperature and 70 ºC for 12 h and 24 h, respectively. The separated membrane from the glass surface was placed in 2 M sulfuric acid for 12 h to adsorb sulfonic acid functional groups. Then the excess acid was removed by washing the membrane with distilled water. The thicknesses of the membranes were fixed at about 70 µm for all tests.

### MEA preparation

To preparation catalyst ink, Pt/C (20 wt% Pt) suspension was obtained by its dispersion in water and isopropyl alcohol by the ultrasonication process. Then, Nafion with a dry weight equal to that of Pt was mixed with the mentioned suspension. Nafion acts as the ionomer binder at the catalyst layer, and it plays a critical role as the ionic conductor in the electrode^34^. Finally, a few drops of glycerol was added and suspended for 10 min in ultrasonic. The catalyst mixture was painted on the carbon clothes until Pt loading reached the desired amount. Drying of the anode and cathode was performed at 80 ºC for 40 min and then at 120 ºC for 60 min. The amount of Pt loading for the electrodes was 0.25 mg cm^−2^. To MEA preparation, the electrodes were placed on two sides of the membrane and then pressed at 120 ºC for 3 min. Then, two graphite plates were connected to the resulting MEA as bipolar plates. The polarization tests were carried out at 80 ºC and 90% relative humidity. The flow rate of H_2_ and O_2_ were selected 120 mL min^−1^ and 300 mL min^−1^, respectively.

### Characterization

In order to characterize the synthesized core–shell nanoparticles, a Bruker Vertex80 instrument was used to obtain their FTIR spectra in the range from 400 to 4000 cm^−1^, and the crystallinity property of the membranes was investigated using an INEL, EQUINOX 3000, X-ray diffractometer.

Thermogravimetric analysis (TGA/SDTA851) was used for the determination of the grafting amount of the polymers (PAMPS and PVBS) on Fe_2_TiO_5_ nanoparticles at the heating rate of 20 °C/min from room temperature to 750 °C. The grafting percentage (G%) was obtained by the following equation, where m_250_ and m_700_ show the mass of the sample at 250 °C and 700 °C, respectively.

2 $$G(\% ) = \frac{{m_{250} - m_{700} }}{{m_{700} }} \times 100$$

The following equation was used for obtaining the polymerization conversion percentage (C), with W_m_ and W_n_ as the weight of monomer and nanoparticles, respectively.

3 $$C(\% ) = \frac{{G(\% ) \times W_{n} }}{{W_{m} }}$$

Besides, a field emission scanning electron microscope (FESEM, TESCAN) was utilized for the determination of the morphology of the nanoparticles and membranes. Energy-dispersive X-ray spectroscopy (EDX, TESCAN) was used as a technique to investigate the dispersion quality of nanoparticles in the nanocomposite membrane. Furthermore, the size of the nanoparticles was studied by using TEM (Zeiss-EM10C). Also, the surface topography of membranes was investigated by atomic force microscopy (AFM, SPA-300HV). An STM-50 testing machine (SANTAM DBBP-Iran) was used for the study of the mechanical properties of the membranes at room temperature and a stretching rate of 2 mm min^−1^.

### Water uptake and swelling

Water uptake (WU) of the membranes (2 cm × 1 cm) was obtained by the following equation:

4 $$WU(\% ) = \frac{{W_{w} - W_{d} }}{{W_{d} }} \times 100$$

where, W_w_ and W_d_ denote the weight of the wet and dry membrane, respectively. For the determination of WU, the samples firstly were dried in an oven at 80 °C for 24 h. Then, they were soaked in deionized water for 12 h and weighed after removal of the surface water of the hydrated membranes. Afterward, the water of the wet membranes was removed by drying in an oven at 80 °C, and then the weight of the dried membranes was obtained again. The length swelling of membrane (MS) was also determined by the below equation.

5 $$MS(\% ) = \frac{{L_{w} - L_{d} }}{{L_{d} }} \times 100$$

where L_w_ and L_d_ indicate the lengths of the wet and dry membrane, respectively.

### Proton conductivity and ion exchange capacity

**Figure 1:**
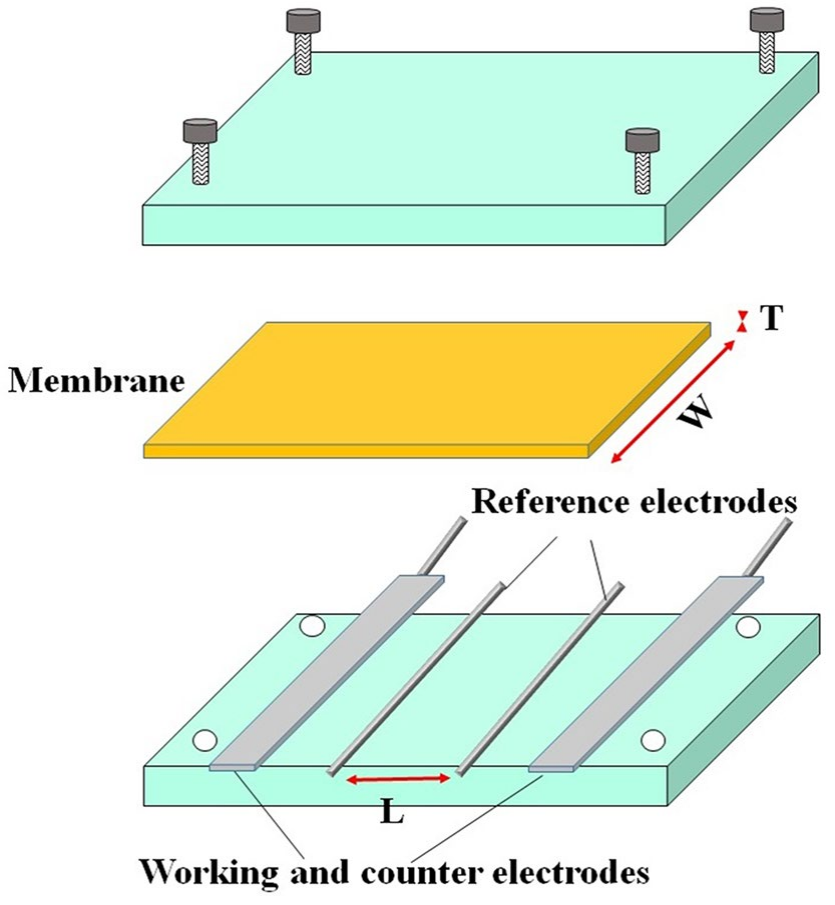
The schematic of proton conductivity measurement cell.

Proton conductivity of the prepared membranes was obtained by the electrochemical impedance spectroscopy (EIS) test via measurement of the ohmic resistance of the membrane at 100% relative humidity. So, proton conductivity values of the membranes were determined from the Nyquist plot. Figure 1 shows a schematic of a cell used for the measurement of proton conductivity by using two platinum plates and two platinum wires. It is good mentioning that the membranes should be prepared for the test by immersing in 2 M H_2_SO_4_ solution for 24 h and then in deionized water for 24 h, respectively.

The proton conductivity (σ (mS cm^−1^)) of the membranes was determined by Eq. (6), where L, W, T, and R are the distance between electrodes (0.6 cm), the width (cm), thickness (cm), and resistance (Ω) of the membrane, respectively.

6 $$\sigma = \frac{L}{RWT}$$

For obtaining the IEC, the membranes needed to be soaked in 2 M H_2_SO_4_ for 24 h in order to substitute all Na^+^ ions with H^+^ ions^35^. In addition, after washing with deionized water, they were dried in an oven. Afterward, the membranes were kept in 1 M NaCl to ensure the substitution of H^+^ ions. Finally, 0.01 M NaOH solution was used for the neutralization of the obtained solutions. The IEC of the prepared membranes was determined using the below equation.

7 $$IEC = \frac{MV}{m}$$

where M, V, and *m* are the concentration of NaOH (M), the volume of consumed NaOH (mL), and the mass of the dry membrane (g), respectively.

### Chemical stability

Fenton's reagent (3% H_2_O_2_ with 4 ppm FeSO_4_) was used for the investigation of the chemical stability of the membranes at 80 °C^36^. For this purpose, a small piece of the samples was weighed and immersed in Fenton's reagent. The chemical stability of the membranes obtained from their residual weight after immersing for 1–4 h.

## Result and discussion

### Characterization of nanoparticles

**Figure 2:**
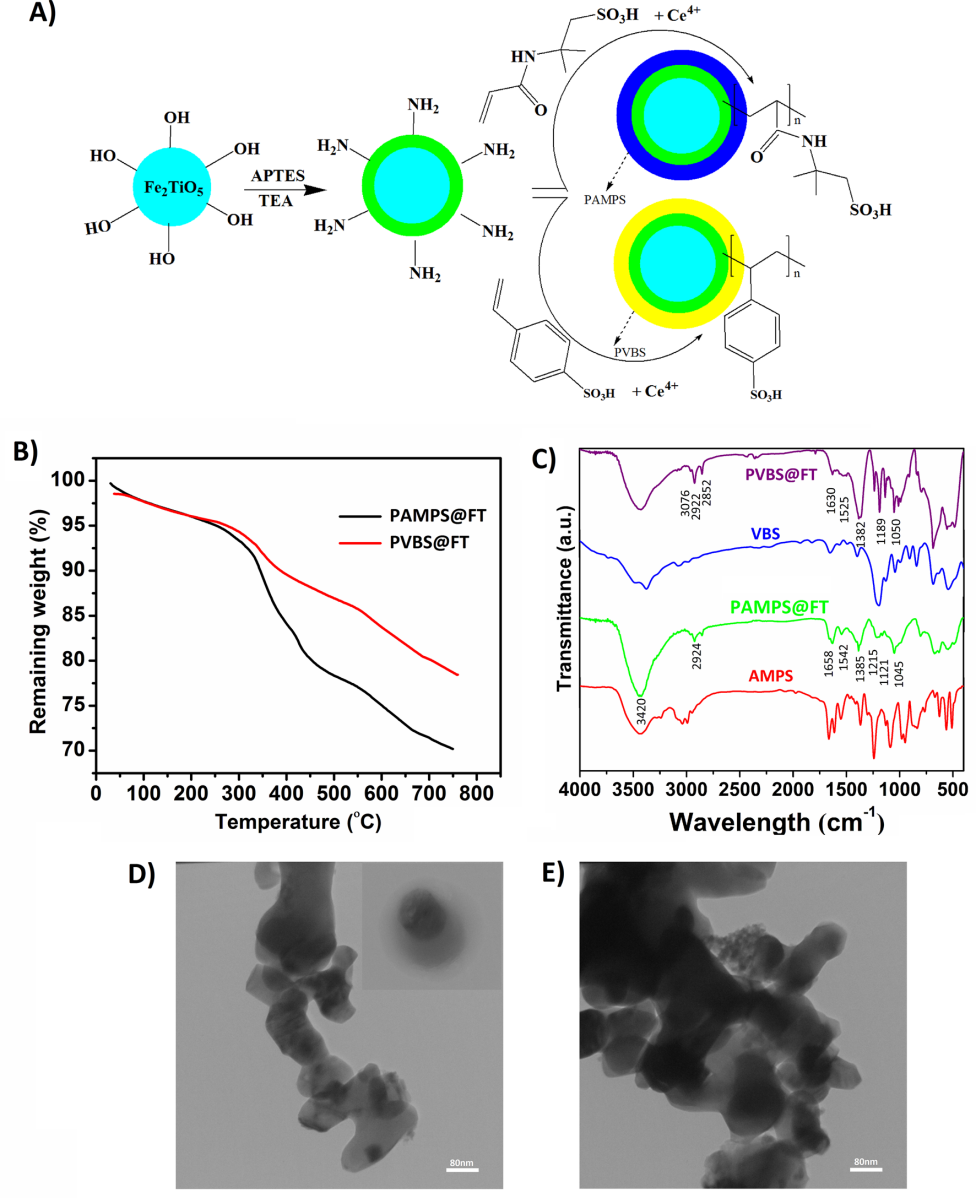
Schematic of polymerization mechanism (**A**), FTIR spectra of PVBS@FT, VBS, PAMPS@FT, and AMPS (**B**), TGA curves of PVBS@FT and PAMPS@FT (**C**), and TEM micrographs of PVBS@FT (**D**) and PAMPS@FT (**E**).

The mechanism of redox polymerization on the surface of nanoparticles was shown in Fig. 2A. Figure 2B shows FT-IR spectra related to PAMPS@FT and PVBS@FT nanoparticles and AMPS and VBS monomers. As can be seen from Fig. 2B, PAMPS polymer has been grafted successfully onto the surface of nanoparticles. Broad peaks around 3422 and 3420 cm^−1^ were ascribed to the hydroxyl group and trapped water which have overlapped with the stretching vibration of the N–H bond. The peak at 1542 cm^−1^ is assigned to the bending vibration of N–H^21^. Furthermore, the existence of the carboxyl group was indicated from the peak at 1658 cm^−1^. Also, the observed peak at 1385 cm^−1^ is assigned to C-N, and the peaks around 1045, 1121, and 1215 cm^−1^ are ascribed to the symmetric and asymmetric vibrations of SO_3_H and stretching vibration of the S=O bond, respectively. Moreover, stretching vibration of CH_2_ and CH_3_ groups are observed around 2853 cm^−1^ and 2924 cm^−1^^21,34^. According to PVBS@FT spectra, peaks around 1050, 1189 cm^−1^ are attributed to the symmetric vibration of SO_3_H and S=O. Absorbance peak at 1382 cm^−1^ is related to bending vibrations of CH_2_ and peaks at 1525 and 1630 cm^−1^ shows the stretching vibration of the aromatic C=C bond. Also, the peaks around 2852 and 2922 cm^−1^ are ascribed to the stretching vibrations of CH_2_ groups. In addition, FT-IR spectra show that PVBS and PAMPS polymers are grafted to the surface of the nanoparticle.

To obtain the amount of grafting percentage of the polymers onto Fe_2_TiO_5_ nanoparticles, the TGA curves were investigated. The related curves are shown in Fig. 2C. Considering the result of TGA analysis, the percentage of grafting and the polymerization conversion percentage are calculated by Eqs. (2) and (3), respectively. The results are shown in Table 1. AMPS monomer shows a higher percentage of grafting in comparison with that of VBS. This behavior can be assigned to the monomer constant rate, possible interactions between functional groups of monomers and nanoparticles or separation behavior (acid strength) of PVBS and PAMPS polyelectrolyte.

**Table 1:** The results of TGA analysis for core–shell nanoparticles.

Sample	Grafting (Wt%)	Conversion (%)	Polymer (mmol/g)	Number of SO_3_H
PAMPS@FT	27.64	2.76	1.33	1.33
PVBS@FT	13.62	1.36	0.74	0.74

TEM images of PAMPS@FT and PVBS@FT nanoparticles were shown in Fig. 2C,D. The TEM images showed a semi-spherical morphology for the hybrid nanoparticles with 70–100 nm size for the nanoparticles based on FT. The layers around the nanoparticles proved that the polymer exists on the surface of the nanoparticles. By attaching PAMPS and PVBS hydrophilic polymers to the surface of nanoparticles, the free energy of the surface of the nanoparticles is decreased. In addition, PAMPS and PVBS monomers have sulfonic acid groups, which add the emulsifying property to the polymer. Hence, the polymer chains that are attached to the surface of nanoparticles prevent aggregating and depositing the reaction zone^37^.

### Structure and morphology of membranes

**Figure 3:**
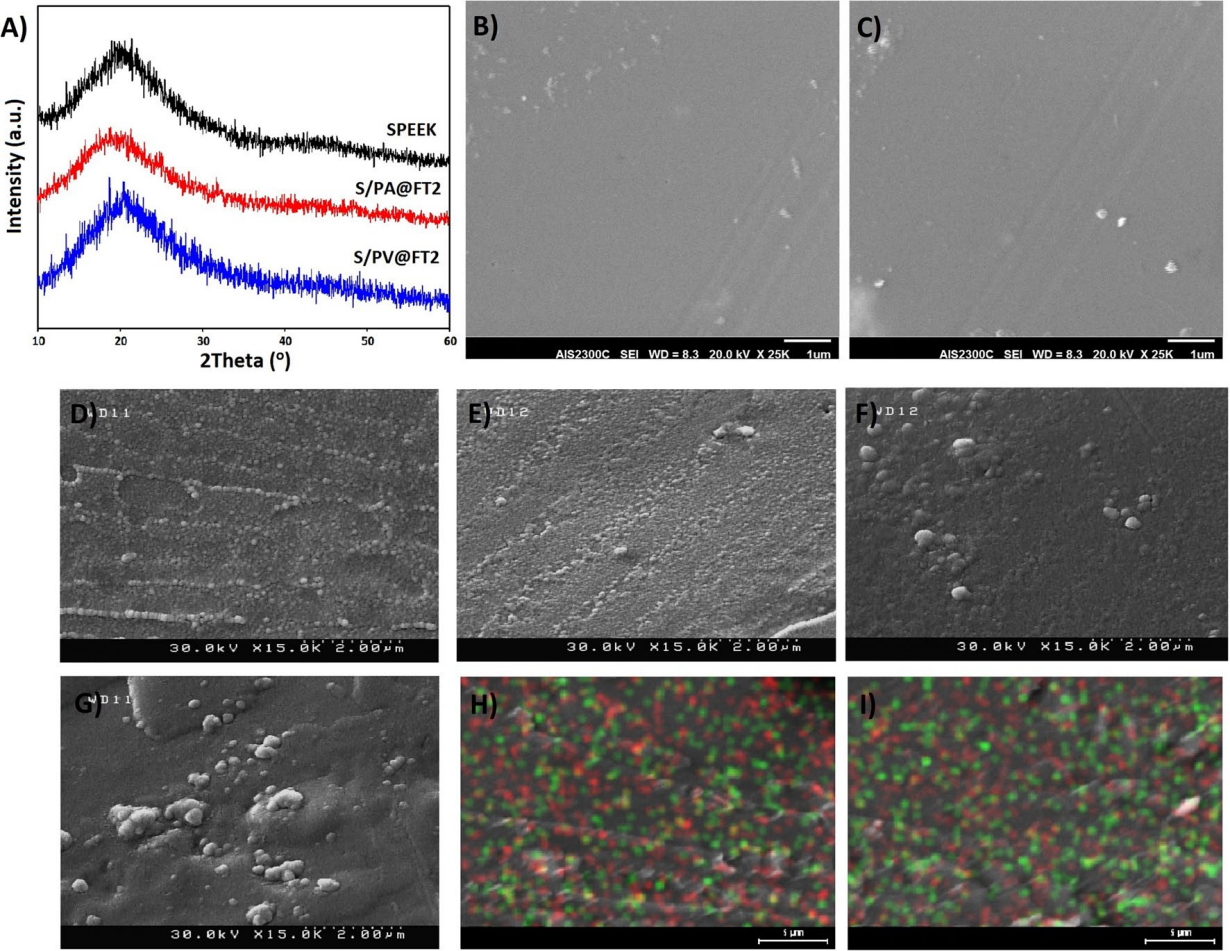
The XRD patterns of SPEEK, S/PA@FT, and S/PV@FT (**A**), the SEM surface images of S/PA@FT2 (**B**) and S/PV@FT2 (**C**), and cross-section FESEM images of S/PA@FT2 (**D**) S/PV@FT2 (**E**), S/PA@FT3 (**F**), and S/PV@FT3 (**G**), and EDX mapping of S/PA@FT2 (**H**) and S/PV@FT2 (**I**). The red points show the Fe element and the green points indicate Ti elements.

The XRD patterns of SPEEK and nanocomposite membranes were shown in Fig. 3A. The crystallinity percentage (X_c_) of the membranes is calculated by X_c_^1/3^ = K_A_/β and is shown in Table 2. In this equation, β is the full width of the peak at the half intensity and K_A_ is a constant equal to 0.24. Based on the obtained results, the crystallinity percentage of the SPEEK membrane was found to be higher in comparison with the nanocomposite membranes. This shows that the nanoparticles have been mixed uniformly in the matrix. Thus the semi-crystalline structure of the SPEEK membrane is more disordered. Disordering in crystalline structure or decrease in the size of the crystal structure is created through the tribulation in crystals with the corporation of the nanoparticles. S/PA@FT membrane shows less crystallinity so that this reduction is related to the amount of PAMPS grafting on the surface of the nanoparticles.

**Table 2:** The crystallinity of the optimized membranes compared to SPEEK.

Membrane	FWHM	Xc (%)
SPEEK	0.1601	3.3687
S/PA@FT2	0.2516	0.8680
S/PV@FT2	0.2465	0.9230

Figure 3B–G show FESEM images of the surface and cross-section of the as-prepared nanocomposite membranes. From Fig. 3B–G, the nanocomposite membranes were synthesized without any structural defects. PAMPS@FT and PVBS@FT nanoparticles have been scattered uniformly through the nanocomposite membrane containing 2wt% of hybrid nanoparticles (Fig. 3D,E). The grafting of PAMPS and PVBS on the surface of the nanoparticles increased the repulsion between the nanoparticles and created uniform scattering of the nanoparticles in the polymer matrix. This happens as a result of the interaction between sulfonic acid groups of PAMPS and PVBS, which are attached to the nanoparticles with sulfonic acid groups of the polymeric matrix. Also, interfacial interactions between the nanoparticles and the polymer matrix can create continuous proton transfer channels. However, there is some agglomeration for membranes containing 3wt% of PAMPS@FT and PVBS@FT nanoparticles (Fig. 3F,G). Besides, EDX mapping of S/PA@FT2 (Fig. 3H) and S/PV@FT2 (Fig. 3I) show the uniform distribution of Fe and Ti elements.

**Figure 4:**
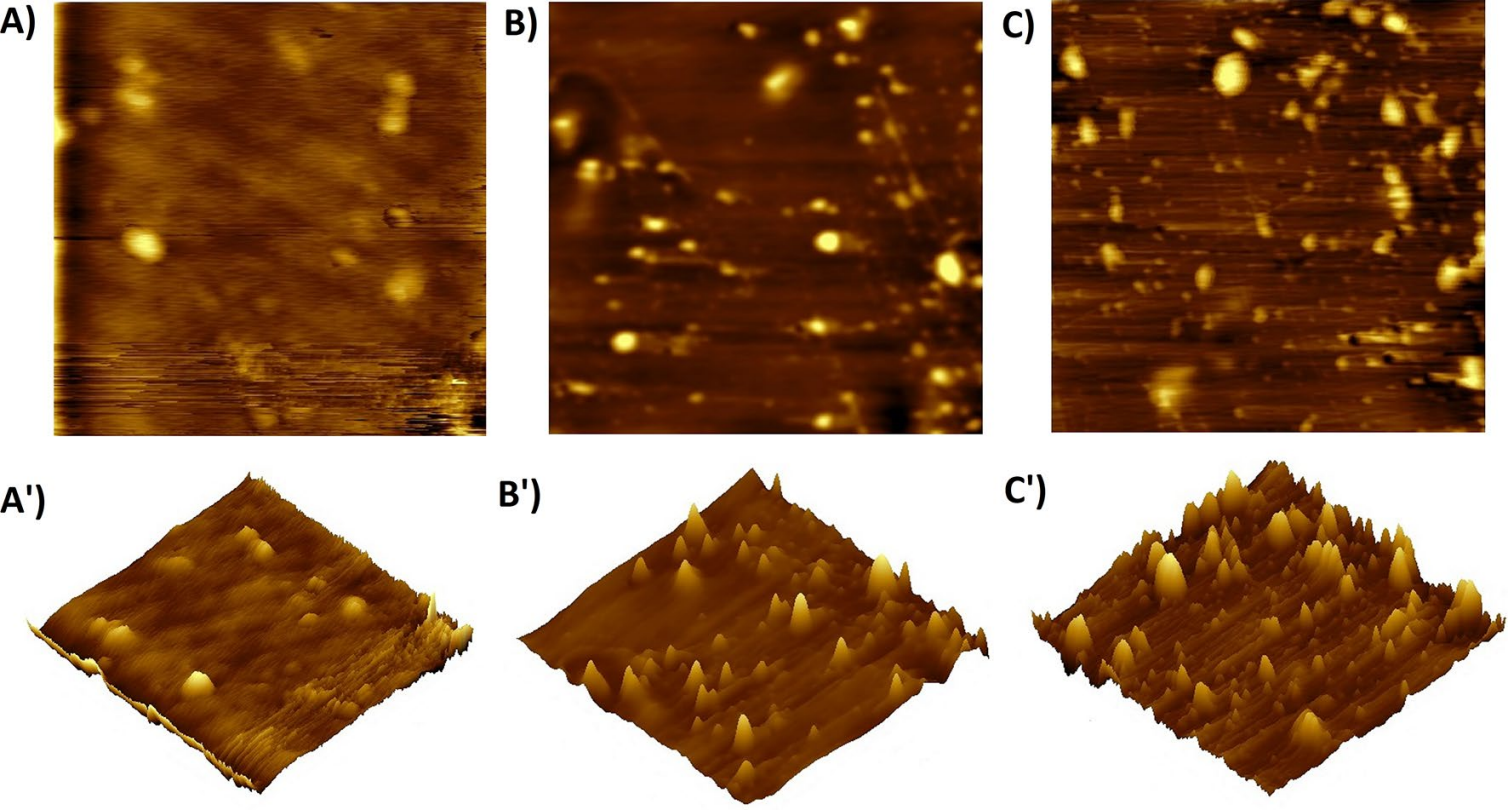
2D and 3D AFM images of the SPEEK (**A**,**A′**), S/PV@FT2 (**B**,**B′**) and S/PA@FT2 (**C**,**C′**).

Figure 4A–C shows the 2D and 3D AFM images of the SPEEK, S/PV@FT2, and S/PA@FT2 membranes. The bright regions of the images are assigned to the hydrophilic area and dark domains refer to the hydrophobic area. Nanocomposite membranes indicate better microphase separations than SPEEK, which attributed to the good dispersion of sulfonated nanoparticles in the membranes. The sulfonated groups create proton conductivity channels in membranes. The size of hydrophilic domains in nanocomposites is higher than that of SPEEK, representing the excellent microphase separation in nanocomposites.

### Water uptake and swelling

**Figure 5:**
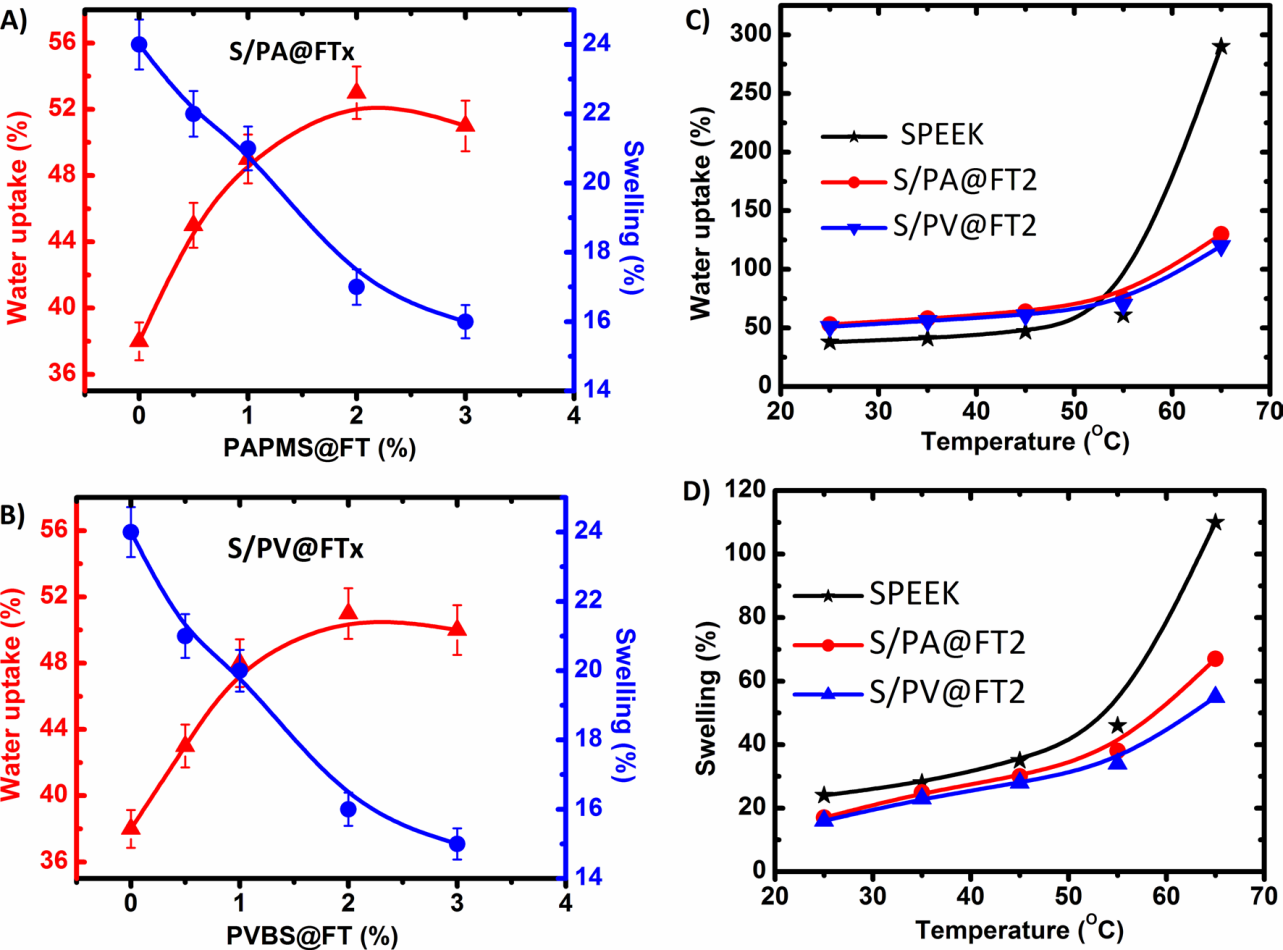
Water uptake and length swelling of S/PA@FTx (**A**) and S/PV@FTx (**B**) membranes versus weight percentage of the nanoparticles and dependence of length swelling (**C**) and water uptake (**D**) of the selected membranes with temperature.

Figure 5A,B shows water uptake and length swelling diagrams of the prepared membranes at ambient temperature. The results showed that an increase in the amount of both nanoparticles up to 2 wt% increases the water uptake of the nanocomposite membranes. This increase is assigned to the hydrophilic property of the grafted sulfonated polymers and the increased volume of water in the interface of the polymer and sulfonated hybrid nanoparticles. But, increasing the amount of PAMPS@FT and PVBS@FT nanoparticles (up to 2 wt%) decreased the water uptake. This reduction is a result of the blocking effect of hybrid nanoparticles, which prevent water molecules to enter the polymeric matrix. The water uptake of the core–shell S/PA@FT membranes was found to be a little more than that of the S/PV@FT membranes, which are attributed to the grafting percentage of PAMPS in comparison to that of PVBS.

Considering Fig. 5A,B, the length swelling values of S/PA@FT and S/PV@FT nanocomposite membranes reduced with the increasing amount of the hybrid nanoparticles. This reduction is related to the interaction between the acid groups of the nanoparticles and those of the polymer. But, the value of the membrane length swelling of S/PA@FT is a little more than that of the S/PV@FT membrane because the PAMPS@FT nanoparticles include lots of PAMPS hydrophilic groups. It is be noted that the swelling of membranes can be reported as length swelling, area swelling and volume swelling^38^. Our reported swelling is the length swelling according to Eq. (5).

Figure 5C,D shows water uptake and length swelling values of the membranes as functions of temperature. In all three membranes with the temperature increase from 25 to 55 °C, water uptake and length swelling values are increased regularly because of the high mobility of polymer chains. For instance, the water uptake of SPEEK and S/PA@FT increased from 38 to 61% and 58% to 75%, respectively. S/PA@FT and S/PV@FT nanocomposite membranes showed higher dimensioned stability in comparison with SPEEK for higher temperatures than 50 °C. This happens because of limiting the polymer chains within the sulfonated hybrid nanoparticles indicating the stability of the membranes in water.

### Proton conductivity and ion exchange capacity

The IEC stands for the amount of ion (proton) exchange groups in the membrane and is named ion exchange capacity. On the other hand, membrane IEC shows the number of hydrophilic groups, which can be ionized and exhibit the acid groups in 1 g of each sample. Regularly, the membranes with high IEC show high proton conductivity because of reduced distance among some groups which can be ionized. IEC data of the nanocomposite membranes are shown in Table 3. With the addition of PAMPS@FT and PVBS@FT hybrid nanoparticles to the SPEEK polymer matrix, the value of IEC is moderately increased. This behavior of IEC is because of the existence of sulfonic acid groups, the higher water uptake of these membranes in comparison with SPEEK, and suitable dispersion of nanoparticles in the matrix leading to more sulfonic acid groups. But, for higher nanoparticle weight percentages (> 2 wt%), IEC experienced a decreasing trend because of the blocking effect of the nanoparticles. Theoretically, it expects that the IEC increases with the addition of sulfonated nanoparticles, but according to our experimental observations, when nanoparticles increase above the critical value, the IEC decreases due to the agglomeration and accumulation of nanoparticles. Agglomeration of nanoparticles can reduce the mobility of proton ions and leads to a decrease in IEC and proton conductivity. Similar results for IEC of nanocomposite membranes were reported by other researchers^39–41^.

**Table 3:** IEC and activation energy of the nanocomposite membranes.

Membrane	SPEEK	S/PA@FT0.5	S/PA@FT1	S/PA@FT2	S/PA@FT3
IEC (meq g^−1^)	1.95	1.97	1.99	2.02	1.99
Ea (kJ mol^−1^)	15.61	11.28	9.91	9.35	9.67

Membrane	–	S/PV@FT0.5	S/PV@FT1	S/PV@FT2	S/PV@FT3
IEC (meq g^−1^)	–	1.96	1.97	1.98	1.97
Ea (kJ mol^−1^)	–	12.26	10.41	9.32	9.46

**Figure 6:**
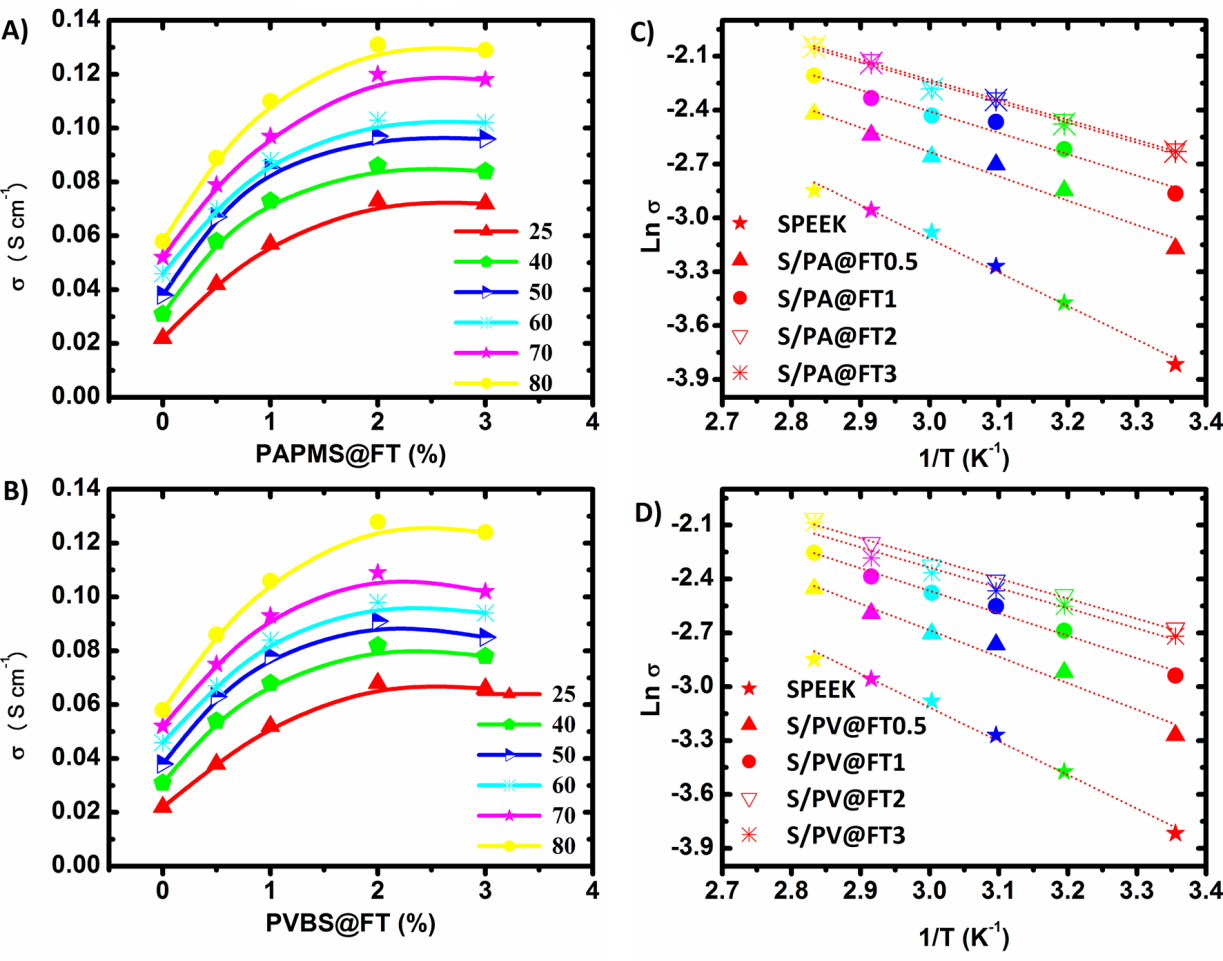
Proton conductivity of S/PA@FTx (**A**) and S/PV@FTx (**B**) membranes versus the percentage of nanoparticles and Arrhenius plots of S/PA@FTx (**C**) and S/PV@FTx (**D**) membranes.

Proton conductivity is an important factor for the evaluation of the performance of PEM. Proton conductivity of the nanocomposite membranes is affected by some parameters like IEC, amounts of water uptake, dispersion of nanoparticles, and the number of nanoparticles in the polymer. The proton conductivity of the membranes based on hybrid nanoparticles and Arrhenius curves were shown in Fig. 6A–D. By the increasing amount of sulfonated hybrid nanoparticles, proton conductivity is also increased. This phenomenon could be explained by increased IEC because it leads to more sites for proton hopping (jumping). Furthermore, the addition of hybrid nanoparticles leads to an increase in the attachment between proton transfer channels and create a simple proton transfer through the membranes. The hybrid nanoparticles have plenty of SO_3_H groups in their surface layers; hence they help to form additional hydrophilic channels. In addition, hydrophilic layers containing PAMPS and PVBS increase the size of hydrophilic channels near the nanoparticle and their existence leads to an increase in proton conductivity and a decrease in the activation energy. But, for nanoparticles amount higher than 2 wt%, as a result of aggregating nanoparticles and blocking the path of proton transfer, proton conductivity was reduced.

Activation energy related to proton transfer is calculated based on the Arrhenius equation and their values are shown in Table 3.

8 $$\sigma = \sigma_{0} \exp ( - E_{a} /kT)$$

where σ, σ_0_, k, and T refer to proton conductivity, pre-exponential factor, the Boltzmann’s constant, and temperature (K), respectively. The activation energy (E_a_) of S/PA@FTx and S/PV@FTx membranes were lower in comparison with that of SPEEK/Fe_2_TiO_5_ and SPEEK/amine-functionalized Fe_2_TiO_5_ nanocomposite membranes reported in our previous work^25^, which means that proton transfer would fall through the vehicle mechanism in these membranes. Furthermore, the performance of the prepared membranes has been compared with five types of nanocomposite membranes (Table 4). As revealed in Table 4, the proton conductivity of our optimum membranes is better than the results of the related literature^42–46^.

**Table 4:** Comparison of proton conductivity of the best prepared membranes with other literatures.

Membrane	Reinforcement	σ (mS cm^−1^)	T (°C)	References
S/PA@FT2	PAPMS@FT2%	131	80	This work
S/PV@FT2	PVBS@FT2%	128	80	This work
SP/SiO_2_-Cloi9%	SiO_2_-Cloi-9%	148.3	100	^42^
SPEEK-SCNF^a^-1	SCNF-1%	128	60	^45^
SPEEK/SBSM^b^	SBSM-15%	28.4	25	^44^
SPEEK/TiO_2_	TiO_2_-7.5%	135	80	^46^
SGO^c^/SPEEK^c^	SGO-5%	55	80	^43^
sPEEK/sPOSS^d^	sPOSS-1.5%	97	80	^47^

^a^Sulfonated carbon nanofiber, ^b^superabsorbent microsphere with imidazole groups, ^c^sulfonated graphene oxide, ^d^sulfonated polyhedral oligomeric silsesquioxane.

### Chemical stability

**Figure 7:**
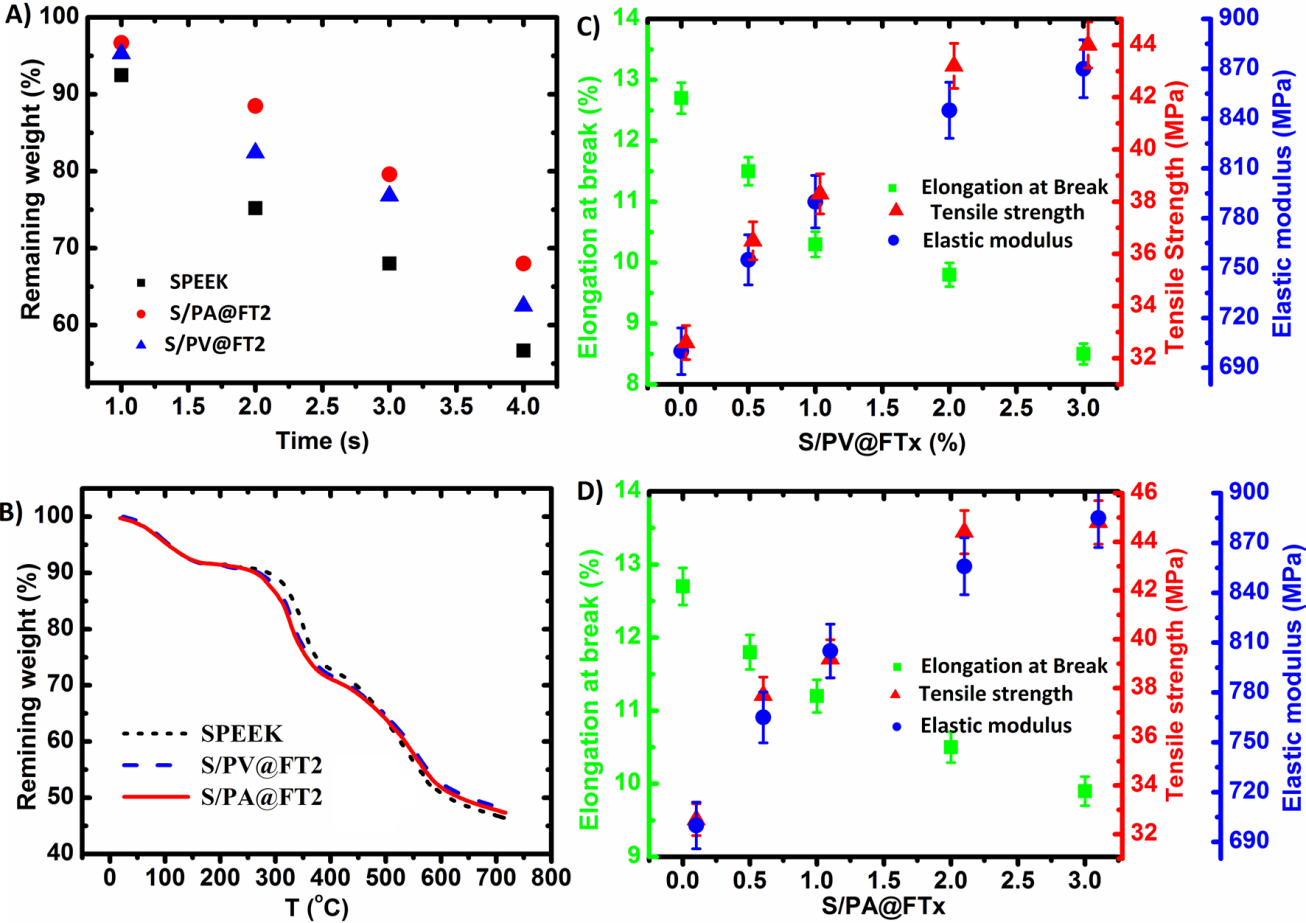
The plots of Fenton test of the selected membranes during 4 h (**A**), TGA curves of the selected membranes (**B**), and the plots of the tensile strength, elastic modulus, and elongation at break for S/PV@FTx (**C**) and S/PA@FTx membranes (**D**).

The Fenton test is a widely-used durability test for PEMs^48^. Chemical stability of the optimum nanocomposite membranes against ^**·**^OH and ^**·**^OOH radicals was studied by Fenton reagent containing 4 ppm FeSO_4_ and 3% H_2_O_2_. The residual weight of membranes during 4 h is shown in Fig. 7A. The result shows that both nanocomposite membranes indicate better stability in comparison with SPEEK against Fenton reagent. Furthermore, the chemical stability of S/PA@FT2 and S/PV@FT2 membranes after 4 h are improved by 16% and 15% in comparison with pristine SPEEK, respectively. This improved chemical stability is because of the interaction between hybrid nanoparticles with sulfonic acid groups and limiting the radical penetration into the membrane.

### Thermal and mechanical stability

The TGA curves related to SPEEK, S/PA@FT, and S/PV@FT membranes were shown in Fig. 7B. Both nanocomposite membranes showed a similar destruction pattern. The destruction step of sulfonic acid groups is in the range of 250 ºC–400 ºC and the demolition of the SPEEK main chains is about 450 ºC. As a result of sulfonated hybrid nanoparticles presence in the SPEEK matrix, S/PA@FT2 and S/PV@FT2 membranes showed lower destruction temperatures in comparison with those of SPEEK. Because PAMPS@FT and PVBS@FT hybrid nanoparticles are included PAMPS and PVBS in their surface layer and these polymers show less destruction temperature rather than SPEEK. But, the decomposition temperature of these membranes is suitably high enough to be applied in fuel cells.

Besides, the IEC of SPEEK and optimized nanocomposite membranes were calculated from TGA results by the following equation:

9 $$IEC_{TGA} = \frac{{(M_{250} - M_{400} )1000}}{{M_{250} \times 81.07}}$$

In this equation, M_250_ and M_400_ refer to the mass of the sample at 250 °C and 400 °C. 81.07 is the molecular weight of SO_3_H. The IEC values of 2.45, 2.60, and 2.66 meq/g were obtained for SPEEK, SP/PV@FT2, and SP/PV@FT2 membranes. Although the IEC values obtained from TGA are different from the obtained values by titration method, the order of IEC change is consistent with each other. This difference may be because in this method the destruction start-up temperature and the end-breakdown temperature of sulfonic groups cannot be calculated quite accurately and may overlap with the degradation of other polymer components.

Mechanical properties of S/PA@FTx and S/PV@FTx membranes such as tensile strength, elastic modulus, and elongation at break are shown in Fig. 7C,D. Both tensile strength and elastic modulus of S/PA@FTx and S/PV@FTx are more than those of the SPEEK membrane and enhanced with an increase in the amount of hybrid nanoparticles. But, elongation at the break of nanocomposite membranes reduces with the increasing amount of hybrid nanoparticles that is due to the reinforcing effect of nanoparticles and mobility limitation of polymer chains. The presence of PAMPS and PVBS polymers on the surface of Fe_2_TiO_5_ nanoparticles increases the interfacial compatibility of polymer and nanoparticles. As a result of hydrogen bonding between SPEEK sulfonic acid groups and Fe_2_TiO_5_ nanoparticles, interfacial compatibility between the polymer and nanoparticles would be improved, and they have made stronger cohesion forces with the polymer matrix. Comparing the sulfonated hybrid nanoparticles shows that PAMPS@FT more effectively improved SPEEK mechanical properties rather than PVBS@FT. The mechanical strength improvement of S/PA@FTx membranes rather than other membranes is ascribed to the more grafting (20.95%) in comparison with that of PVBS (12.82%) and as a result of more specific interactions among the acid functional groups of PAMPS and SPEEK. In terms of tensile strength, S/PA@FT2 and S/PV@FT2 membranes showed the most one (44.8 MPa and 44 MPa, respectively), which are 37.4% and 35% higher than that of SPEEK membrane. As the percentage of the hybrid nanoparticles increases to higher than 2 wt%, the increased slope of tensile strength reduces, which probably is due to the aggregating of nanoparticles.

### Proton conductivity stability and fuel cell performance

**Figure 8:**
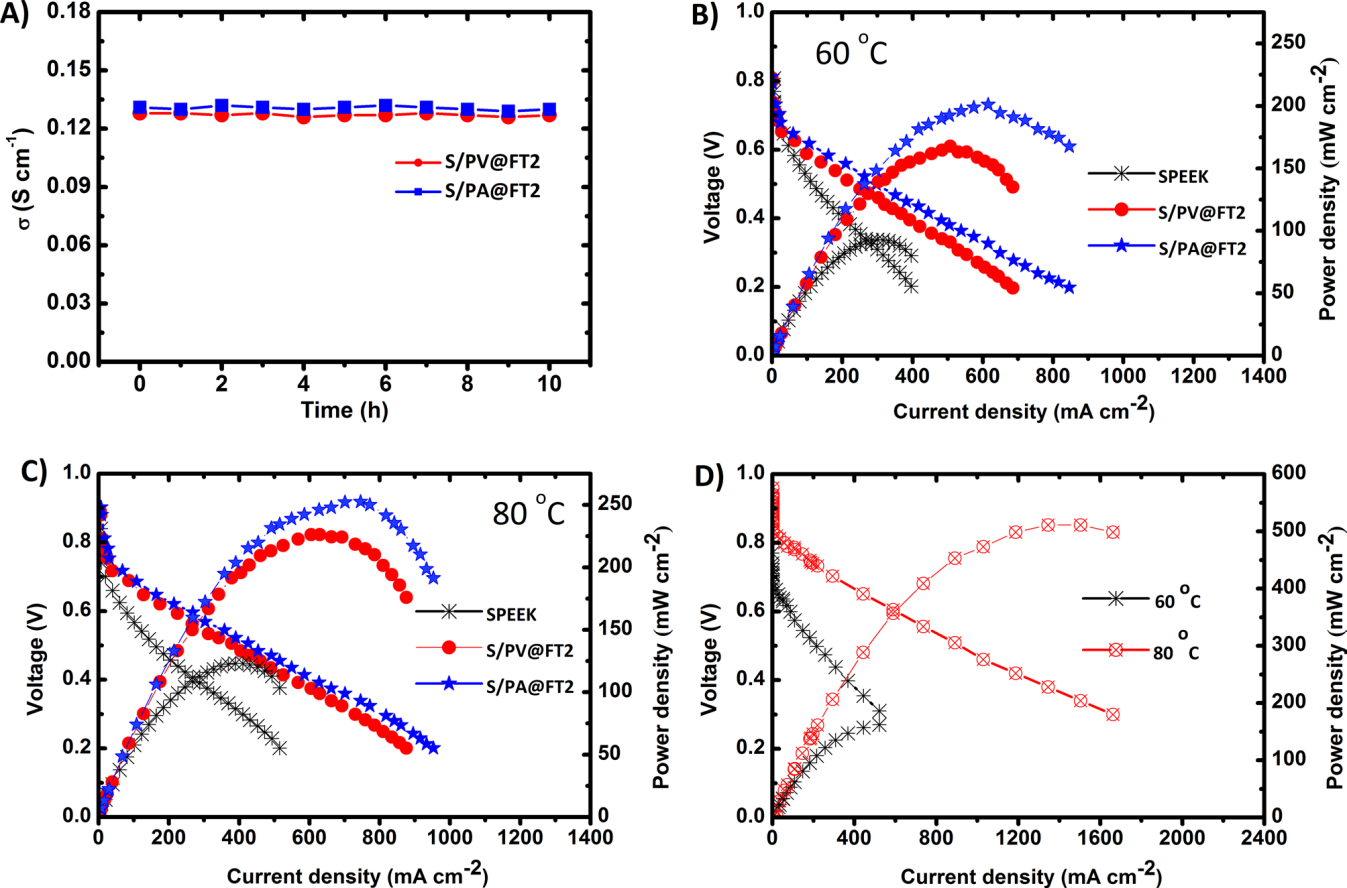
Proton conductivity stability of S/PA@FT2, and S/PV@FT2 for 10 h (**A**), polarization plots of SPEEK, S/PA@FT2, and S/PV@FT2 at 60 °C (**B**) and 80 °C (**C**), and polarization curves of Nafion 117 at different temperatures (**D**).

Proton conductivity stability is an important parameter for evaluating the stability and performance of membranes in fuel cells. Thus, the time-stability of proton conductivity of the optimized membranes were evaluated for 10 h at 80 °C, as shown in Fig. 8A. The proton conductivities of SP/PA@FT2 and SP/PV@FT2 membranes almost constant after 10 h. It outstanding the important role of sulfonated nanoparticles in the stability of proton conductivity.

PEMFC performance tests of MEAs including SPEEK, S/PA@FT2, and S/PV@FT2 membranes were investigated at 60 ºC and 80 ºC, and the results are shown in Fig. 8B,C. The fuel cell performance of all SPEEK-based membranes was increased with temperature from 60 to 80 ºC. The open-circuit voltage (OCV) at 80 ºC for SPEEK, S/PA@FT2, and S/PV@FT2 membranes are 0.84, 0.87, and 0.89 V, respectively. The higher OCV of nanocomposite membranes is attributed to the lower crossover of fuels in the membrane. The maximum power density for S/PA@FT2 and S/PV@FT2 membranes obtained 247 mW cm^−2^ and 226 mW cm^−2^, respectively, which are about 100% and 85% higher than the power density of MEA including SPEEK (122 mW cm^−2^). Figure 8D displays the fuel cell performance for commercial Nafion 117 membrane for comparison in the temperatures of 60 °C and 80 °C. From Fig. 8D, the fuel cell performance of the Nafion 117 membrane at 80 ºC was higher than that of 60 ºC. Although the fuel cell performance of the Nafion 117 membrane (511 mW cm^−2^ at 80 °C) is higher than the as-prepared nanocomposite membranes, however, these nanocomposites have good potential for application in fuel cells.

## Conclusion

In this work, the functionalization of Fe_2_TiO_5_ by SO_3_H-containing polymers, core/shell nanoparticles, and their consequent incorporation into a SPEEK matrix was described to fabricate new nanocomposite membranes. The results showed that the membrane properties such as water uptake, dimensional stability, and proton conductivity improved. In addition, S/PA@FT2 and S/PV@FT2 exhibited the maximum proton conductivity of 0.222 and 0.209 S cm^−1^ at 80 °C, respectively. The sulfonic acid core/shell nanoparticles, as a proton-conducting additive, create continuous proton transfer channels, increase proton conductivity, and enhance ion exchange of membranes because of extra sulfonic acid groups in the matrix of the prepared nanocomposite membrane. Also, the maximum power densities of 247 mW cm^−2^ and 226 mW cm^−2^ were obtained for single cells including S/PA@FT2 and S/PV@FT2 membranes at 80 °C, respectively. So, the results showed that the prepared membranes are very promising as proton exchange membranes in PEMFCs.

## References

[1] Beydaghi H (2020). Enhancing the performance of poly(phthalazinone ether ketone)-based membranes using a new type of functionalized TiO_2_ with superior proton conductivity. Ind. Eng. Chem. Res..

[2] Pei H (2019). Performance improvement in a proton exchange membrane fuel cell with separated coolant flow channels in the anode and cathode. Energy Convers. Manag..

[3] Beydaghi, H. *et al.* Functionalized metallic transition metal dichalcogenide (TaS2) for nanocomposite membrane in direct methanol fuel cells. *J. Mater. Chem. A.*10.1039/D0TA11137F (2021)

[4] Xu G (2020). Non-destructive fabrication of Nafion/silica composite membrane via swelling-filling modification strategy for high temperature and low humidity PEM fuel cell. Renew. Energy.

[5] Cui Y (2018). Porous silicon-aluminium oxide particles functionalized with acid moieties: An innovative filler for enhanced Nafion-based membranes of direct methanol fuel cell. J. Power Sources.

[6] Prapainainar P (2019). Incorporating graphene oxide to improve the performance of Nafion-mordenite composite membranes for a direct methanol fuel cell. Int. J. Hydrogen Energy.

[7] Haragirimana A (2019). Four-polymer blend proton exchange membranes derived from sulfonated poly (aryl ether sulfone) s with various sulfonation degrees for application in fuel cells. J. Membr. Sci..

[8] Salarizadeh P (2016). Surface modification of Fe_2_TiO_5_ nanoparticles by silane coupling agent: Synthesis and application in proton exchange composite membranes. J. Colloid Interface Sci..

[9] Salarizadeh P (2019). Enhanced properties of SPEEK with incorporating of PFSA and barium strontium titanate nanoparticles for application in DMFCs. Int. J. Energy Res..

[10] Beydaghi H (2017). Novel proton exchange membrane nanocomposites based on sulfonated tungsten trioxide for application in direct methanol fuel cells. Polymer.

[11] Hooshyari K (2015). Fabrication BaZrO_3_/PBI-based nanocomposite as a new proton conducting membrane for high temperature proton exchange membrane fuel cells. J. Power Sources.

[12] He G (2014). Enhancing water retention and low-humidity proton conductivity of sulfonated poly (ether ether ketone) composite membrane enabled by the polymer-microcapsules with controllable hydrophilicity–hydrophobicity. J. Power Sources.

[13] Li Z (2020). Addition of modified hollow mesoporous organosilica in anhydrous SPEEK/IL composite membrane enhances its proton conductivity. J. Membr. Sci..

[14] Tripathy M, Kumar PS, Deshpande AP (2017). Molecular structuring and percolation transition in hydrated sulfonated poly (ether ether ketone) membranes. J. Phys. Chem. B.

[15] Elwan HA, Mamlouk M, Scott KA (2020). A review of proton exchange membranes based on protic ionic liquid/polymer blends for polymer electrolyte membrane fuel cells. J. Power Sources.

[16] Gahlot S, Kulshrestha V (2020). Graphene based polymer electrolyte membranes for electro-chemical energy applications. Int. J. Hydrogen Energy.

[17] Marani D (2010). Titania nanosheets (TNS)/sulfonated poly ether ether ketone (SPEEK) nanocomposite proton exchange membranes for fuel cells. Chem. Mater..

[18] Lade H (2017). Sulfonated poly (arylene ether sulfone) nanocomposite electrolyte membrane for fuel cell applications: A review. Int. J. Hydrogen Energy.

[19] Beydaghi H (2015). Novel nanocomposite membranes based on blended sulfonated poly (ether ether ketone)/poly (vinyl alcohol) containing sulfonated graphene oxide/Fe_3_O_4_ nanosheets for DMFC applications. RSC Adv..

[20] Bagheri A (2019). The effect of adding sulfonated SiO_2_ nanoparticles and polymer blending on properties and performance of sulfonated poly ether sulfone membrane: Fabrication and optimization. Electrochim. Acta.

[21] Salarizadeh P (2019). Novel proton exchange membranes based on proton conductive sulfonated PAMPS/PSSA-TiO_2_ hybrid nanoparticles and sulfonated poly (ether ether ketone) for PEMFC. Int. J. Hydrogen Energy.

[22] Hooshyari K (2019). Nanocomposite membranes with high fuel cell performance based on sulfonated poly(1,4-phenylene ether ether sulfone) and ytterbium/yttrium doped-perovskite nanoparticles. J. Electrochem. Soc..

[23] Javanbakht M (2014). Novel PVA/La_2_Ce_2_O_7_ hybrid nanocomposite membranes for application in proton exchange membrane fuel cells. Iran. J. Hydrogen Fuel Cell.

[24] Salarizadeh P, Javanbakht M, Pourmahdian S (2015). Fabrication and physico-chemical properties of iron titanate nanoparticles based sulfonated poly(ether ether ketone) membrane for proton exchange membrane fuel cell application. Solid State Ionics.

[25] Salarizadeh P (2016). Influence of amine-functionalized iron titanate as filler for improving conductivity and electrochemical properties of SPEEK nanocomposite membranes. Chem. Eng. J..

[26] Hooshyari K (2020). High temperature membranes based on PBI/sulfonated polyimide and doped-perovskite nanoparticles for PEM fuel cells. J. Membr. Sci..

[27] Moradi M (2016). Experimental study and modeling of proton conductivity of phosphoric acid doped PBI-Fe_2_TiO_5_ nanocomposite membranes for using in high temperature proton exchange membrane fuel cell (HT-PEMFC). Int. J. Hydrogen Energy.

[28] Kim DJ (2016). Characterization of the sulfonated PEEK/sulfonated nanoparticles composite membrane for the fuel cell application. Int. J. Hydrogen Energy.

[29] Rao Z, Tang B, Wu P (2017). Proton conductivity of proton exchange membrane synergistically promoted by different functionalized metal–organic frameworks. ACS Appl. Mater. Interfaces..

[30] Wei Y (2017). Modified nanocrystal cellulose/fluorene-containing sulfonated poly (ether ether ketone ketone) composites for proton exchange membranes. Appl. Surf. Sci..

[31] Zhang J (2017). Ion-exchange-induced selective etching for the synthesis of amino-functionalized hollow mesoporous silica for elevated-high-temperature fuel cells. ACS Appl. Mater. Interfaces..

[32] Qiu X (2017). Poly(2,5-benzimidazole)-grafted graphene oxide as an effective proton conductor for construction of nanocomposite proton exchange membrane. ACS Appl. Mater. Interfaces..

[33] Salarizadeh P (2013). Preparation, characterization and properties of proton exchange nanocomposite membranes based on poly (vinyl alcohol) and poly (sulfonic acid)-grafted silica nanoparticles. Int. J. Hydrogen Energy.

[34] Lopez-Haro M (2014). Three-dimensional analysis of Nafion layers in fuel cell electrodes. Nat. Commun..

[35] Sahin A (2018). The development of Speek/Pva/Teos blend membrane for proton exchange membrane fuel cells. Electrochim. Acta.

[36] Gashoul F, Parnian MJ, Rowshanzamir S (2017). A new study on improving the physicochemical and electrochemical properties of SPEEK nanocomposite membranes for medium temperature proton exchange membrane fuel cells using different loading of zirconium oxide nanoparticles. Int. J. Hydrogen Energy.

[37] Kang T, Jang I, Oh S-G (2016). Surface modification of silica nanoparticles using phenyl trimethoxy silane and their dispersion stability in N-methyl-2-pyrrolidone. Colloids Surf. A.

[38] Zhang N (2014). Quaternized poly(ether ether ketone)s doped with phosphoric acid for high-temperature polymer electrolyte membrane fuel cells. J. Mater. Chem. A.

[39] Simari C (2020). Highly-performing and low-cost nanostructured membranes based on polysulfone and layered doubled hydroxide for high-temperature proton exchange membrane fuel cells. J. Power Sources.

[40] Sivasankaran A, Sangeetha D, Ahn Y-H (2016). Nanocomposite membranes based on sulfonated polystyrene ethylene butylene polystyrene (SSEBS) and sulfonated SiO_2_ for microbial fuel cell application. Chem. Eng. J..

[41] Sugumar M, Dharmalingam S (2020). Statistical optimization of process parameters in microbial fuel cell for enhanced power production using sulphonated polyhedral oligomeric silsesquioxane dispersed sulphonated polystyrene ethylene butylene polystyrene nanocomposite membranes. J. Power Sources.

[42] Charradi K (2019). Silica/montmorillonite nanoarchitectures and layered double hydroxide-SPEEK based composite membranes for fuel cells applications. Appl. Clay Sci..

[43] Kumar R, Mamlouk M, Scott K (2014). Sulfonated polyether ether ketone–sulfonated graphene oxide composite membranes for polymer electrolyte fuel cells. RSC Adv..

[44] Liu W (2020). New sulfonated poly (ether ether ketone) composite membrane with the spherical bell-typed superabsorbent microspheres: Excellent proton conductivity and water retention properties at low humidity. J. Power Sources.

[45] Liu X (2017). Electrospun multifunctional sulfonated carbon nanofibers for design and fabrication of SPEEK composite proton exchange membranes for direct methanol fuel cell application. Int. J. Hydrogen Energy.

[46] Salarizadeh P, Javanbakht M, Pourmahdian S (2017). Enhancing the performance of SPEEK polymer electrolyte membranes using functionalized TiO_2_ nanoparticles with proton hopping sites. RSC Adv..

[47] Kim S-W, Choi S-Y, Rhee H-W (2018). A novel sPEEK nanocomposite membrane with well-controlled sPOSS aggregation in tunable nanochannels for fast proton conduction. Nanoscale.

[48] Tsuneda T (2020). Fenton reaction mechanism generating no OH radicals in Nafion membrane decomposition. Sci. Rep..

